# Identification of Glyceraldehyde 3-Phosphate Dehydrogenase Sequence and Expression Profiles in Tree Shrew (*Tupaia belangeri*)

**DOI:** 10.1371/journal.pone.0098552

**Published:** 2014-06-02

**Authors:** Yu Zheng, Qihui Wang, Chenxia Yun, Yingjun Wang, Wanli W. Smith, Jing Leng

**Affiliations:** 1 Department of Microbiology and Immunology, Guangxi University of Chinese Medicine, Nanning, Guangxi, China; 2 Department of Immunology, Guangxi Medical University, Nanning, Guangxi, China; 3 Guangxi University of Chinese Medicine, Nanning, Guangxi, China; 4 Department of Pharmaceutical Sciences, University of Maryland School of Pharmacy, Baltimore, Maryland, United States of America; Max F. Perutz Laboratories, Austria

## Abstract

The tree shrews (*Tupaia belangeri*) diverged from the primate order (Primates) and are classified as *Scandentia*, a separate taxonomic group of mammals. The tree shrew has been suggested to use an animal model to study human disease but the genomic sequences of tree shrew is largely unidentified. Here we identified the full-length cDNA sequence of a housekeeping gene, Glyceraldehyde 3-phosphate Dehydrogenase (GAPDH), in tree shrew. We further constructed a phylogenetic family tree base on GAPDH molecules of various organisms and compared GAPDH sequences with human and other small experimental animals. These study revealed that tree shrew was closer to human than mouse, rat, rabbit and guinea pig. The Quantitative Reverse Transcription PCR and western blot analysis further demonstrated that GAPDH expressed in various tissues in tree shrew as a general conservative housekeeping proteins as in human. Our findings provide the novel genetic knowledge of the tree shrew and strong evidences that tree shrew can be an experimental model system to study human disorders.

## Introduction

The tree shrews (*Tupaia belangeri*, family *Tupaiidae*) are small mammals native to the tropical forests and plantation areas, broadly distributed across Southeast Asia [1]. It probably diverged from the primate order (*Primates*) about 85 million years ago, which are now widely classified as a separate taxonomic group of mammals (*Scandentia*) [2], [3]. Consequently, tree shrew is a potentially useful animal model for some human diseases because of closer phylogenetic relationship with human [2]. In last decades, tree shrews have been developed as experimental animal and easy to grow in a laboratory [4]–[7]. The number of literatures which tree shrews are used as an experimental animal model in amount of disease is increasing, because of their well-developed visual system, higher-ratio of brain to body mass, or infection of species-especially pathogens, such as hepatitis virus and influenza virus [7]–[11]. Currently, tree shrew models are mainly used for research into the nervous, visual systems, and viral infection disease [12].

However, only a few tree shrew genes have the full-length sequences. In the Genbank database, there are only around 200 molecules with coding information (partial or complete cds). Moreover, there are only a few antibodies and detecting kits available for studying tree shrew. It is difficult to detect and characterize gene and protein expression profile without sequence information and species-especially antibodies. Due to those reasons, it is narrow the wide application of tree shrew as a model for diseases' mechanism research.

Housekeeping gene is a group of typically constitutive genes that are required for the maintenance of basic cellular function. Some housekeeping genes are expressed at relatively constant levels in most physiological situations; others may vary according to experimental conditions [13]–[15]. Therefore, housekeeping genes are generally used as internal reference to normalize target genes in many examine methods, such as quantitative real-time reverse transcription polymerase chain reaction (qRT-PCR), western immunoblotting, immunohistochemistry, and so on. An ideal housekeeping gene as internal reference should be widely stably expressed within all cells of an organism under normal and pathophysiological conditions [14], [16]. To examines of human samples, some housekeeping genes are frequently used as internal reference, which also named endogenous control genes, such as Glyceraldehyde-3-Phosphate Dehydrogenase (GAPDH), β-actin (ACTB), 18S ribosomal RNA (18S rRNA), heat-shock protein-90 (Hsp90), β-2-microglobulin, β-tubulin and others [13]–[15]. However, for tree shrew, most of their sequences are still unknown.

GAPDH playing an important role in both of glycolysis and nuclear functions, respectively, given that is has both glyceraldehyde-3-phosphate dehydrogenase and nitrosylase. GADPH also is an important enzyme in the process of glycolysis and gluconeogenesis, whose cycles occur in the cytoplasm. It is responsible for catalyzing the reversible conversion of glyceraldehyde 3-phosphate (GAP) and inorganic phosphate into 1,3-bisphosphoglycerate (1,3-BPG) with a three-step reaction [17]–[19]. Moverover, GAPDH has been implicated it is involved in several non-metabolic processes, including transcription activation, ER to Golgi vesicle shuttling, initiation of apoptosis, and axoplasmic transport, or fast axonal [20], [21]. Thereby, GAPDH was considered it is experssed in every cell, tissue, and organ. It is one of “housekeeping” molecules that were used as an internal reference to correct the potential error of RNA/cDNA loading, and variation of reverse transcription efficiency. GAPDH is widely considered as a molecule that is expressed at relatively constant levels in most situations. However, some studies disagreed to this [22]–[24].

In this study, we identified full-length cDNA sequence of tree shrew GAPDH (tsGAPDH) gene. Using this sequence, we construct the genetic family tree and study the GAPDH expression profiles in tree shrew. These studies provide the novel genetic and proteomic knowledge of tree shrew, and the strong evidence that tree shrew can be a potential animal model to study human disorders.

## Results

### Identification the full length GAPDH sequence of tree shrew

We identified full-length GAPDH's cDNA in three individual tree shrew specimens. All sequence of three samples is totally same. It is 1296 bp in length and codes a protein with 333 amino acids. The number of amino acid is similar to human's (335), and equal to mouse's (333), rat's (333), rabbit(333), guinea pig (333). The full-length amino acid sequence of tsGAPDH was deduced base on the tsGAPDH cDNA sequence. We deposited the tsGAPDH cDNA and amino acid sequences in GenBank (NIH) with the accession number KC215182. To our knowledge, this is the first time to report a full-length sequence of tsGAPDH.

### GAPDH Phylogenetic Analysis

**Figure 1:**
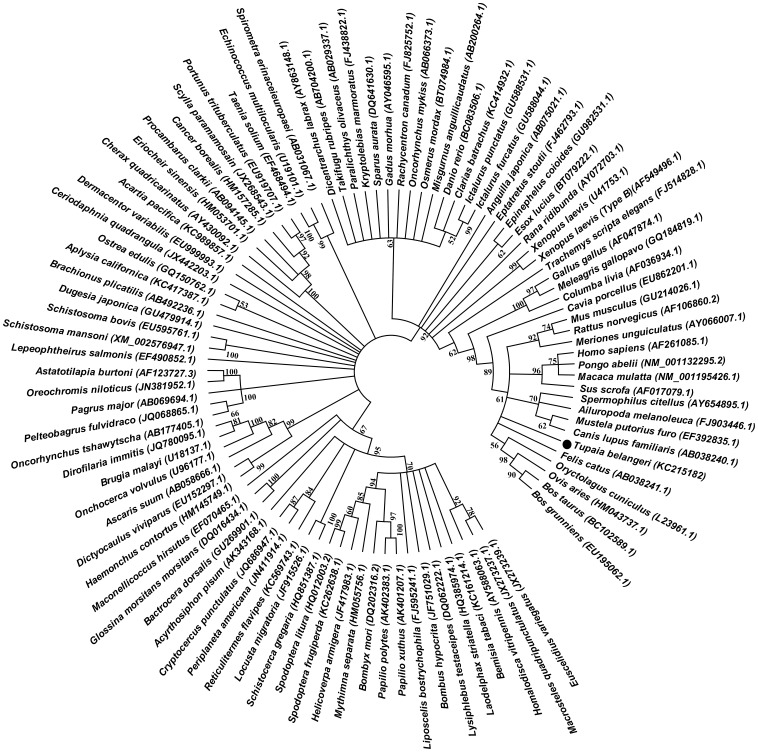
Phylogenetic analysis of GAPDH. The Phylogenetic tree is based on deduced full-length amino acid sequences of GAPDH and constructed with the neighbor-joining method. Only bootstrap values over 50% are shown.

With the full length sequence of tsGAPDH from our studies, we were able to construct a phylogenetic tree (Fig.1) using the genetic database from GenBank. In our diagram of phylogenetic tree, *Feliformia* (cat), *Lagomorpha* (rabbit) and *Caniformia* (dog) as the closer specie to the tree shrew than *Primates*; and tree shrew is closer to *Primates* than *Lagomorpha* (Rabbit) and *Rodentia* (mouse, rat, and guinea pig) those were generally used as experimental animals. This is in consistent with previous studies in tree shrew MHC class I gene [25], [26].

**Figure 2:**
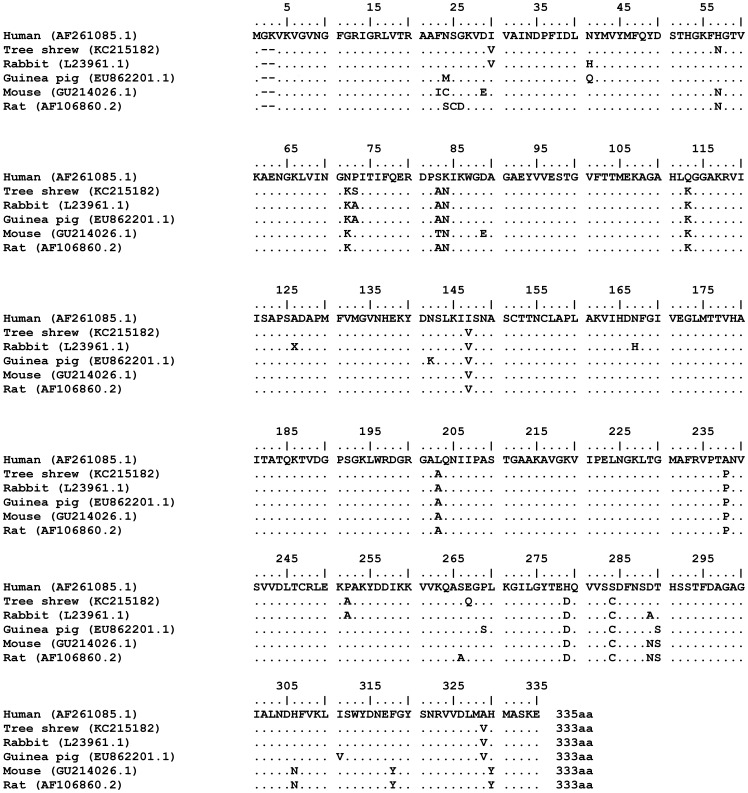
Alignment of GAPDH. Comparison of the deduced full-length amino acid sequences of GAPDH among human, tree shrew, mouse, rat, rabbit and guinea pig. Identical amino acids are shown by a dot, gaps are represented by a dash.

To further study the relations between tree shrew and other species, we compared the sequence of tsGAPDH, with human (*Homo sapiens*) and other small experimental animals which included mouse (*Mus musculus*), rat (*Rattus norvegicus*), rabbit (*Oryctolagus cuniculus*) and guinea pig (*Cavia porcellus*). The multiple sequence alignment (MSA) result (Fig.2) and MSA scores (Table 1) showed that there were 17 different amino acids in GAPDH between tree shrew and human. This difference was less than those of human to mouse (21), to rat (21), to rabbit (19), or to guinea pig (19) as described previously. The tsGAPDH was up to 95.2% of homology with human GAPDH (Table 1).

**Table 1:** MSA Scores of GAPDH.

	human	tree shrews	mouse	rat	guinea pig	Rabbit
**human**	—					
**tree shrews**	95.20	—				
**mouse**	93.99	95.50	—			
**rat**	93.99	95.80	97.60	—		
**guinea pig**	93.99	96.70	95.20	95.50	—	
**Rabbit**	94.59	97.90	94.59	94.89	96.70	—

### Expression-Profile of tsGAPDH Gene in Tree Shrew Tissues

**Figure 3:**
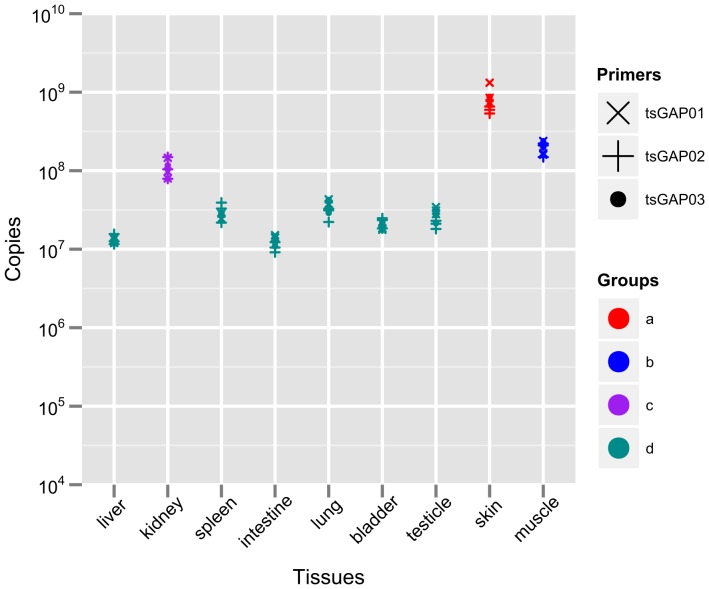
Expression-Profile Measurement of Tree shrew GAPDH Gene. Three pair of primers for qRT-PCR were designed to measure tsGAPDH mRNA expression in various tree shrew tissues. Data are shown in a point chart and grouped according to ANOVA (p<0.05).

To further study the tsGAPDH expression pattern in tree shrew, absolute qRT-PCR was used to measure amount of tsGAPDH mRNA in various tissues. We designed 3 pairs of primers (Table 2) to measure tsGAPDH. The amplification efficiencies of 3 pair of primers were close and various. All examined tissues was expressed GAPDH gene and the amount of GAPDH mRNA was relatively constant in most of tissues detected by all primer pairs. The expression of GAPDH in various tissues were compared by One-Way Analysis of Variance (ANOVA) followed by Duncan's Multiple Range Test, and the significance level was set at P = 0.05 (Result S1). According to this statistical analysis, expression of GAPDH can be grouped to 4 groups, which marked as a, b, c, d (Fig.3). Amount of GAPDH in the same group are not significantly different.

**Table 2:** Primers used in this study.

Primer	Sequence	Application
tsGAP-GSP3RACE	GGCCGTGGGGCTGCCCAGAACA	3'-RACE amplification
tsGAP-GSP5RACE	TGCTTCACCACCTTCTTGATGTCA	5'-RACE amplification
tsGAP-STDf	GATGTCGTGGCCATCAACGACCC	standard plasmid construction
tsGAP-STDr	ACTCCACAACATACTCGGCACC	standard plasmid construction
tsGAP01 (efficeiency 118%)	Forward: TCATTGACCTGAACTACAT Reverse: GAAGATGGTGATGGACTT	qRT-PCR
tsGAP02 (effieiency 102%)	Forward: CCTGAACTACATGGTCTAC Reverse: GAAGATGGTGATGGACTT	qRT-PCR
tsGAP03 (effieiency 118%)	Forward: CGGGAAGTCCATCACCATCT Reverse: CCACAACATACTCGGCACCA	qRT-PCR

### Expression-pattern of GAPDH in Tree Shrew

**Figure 4:**
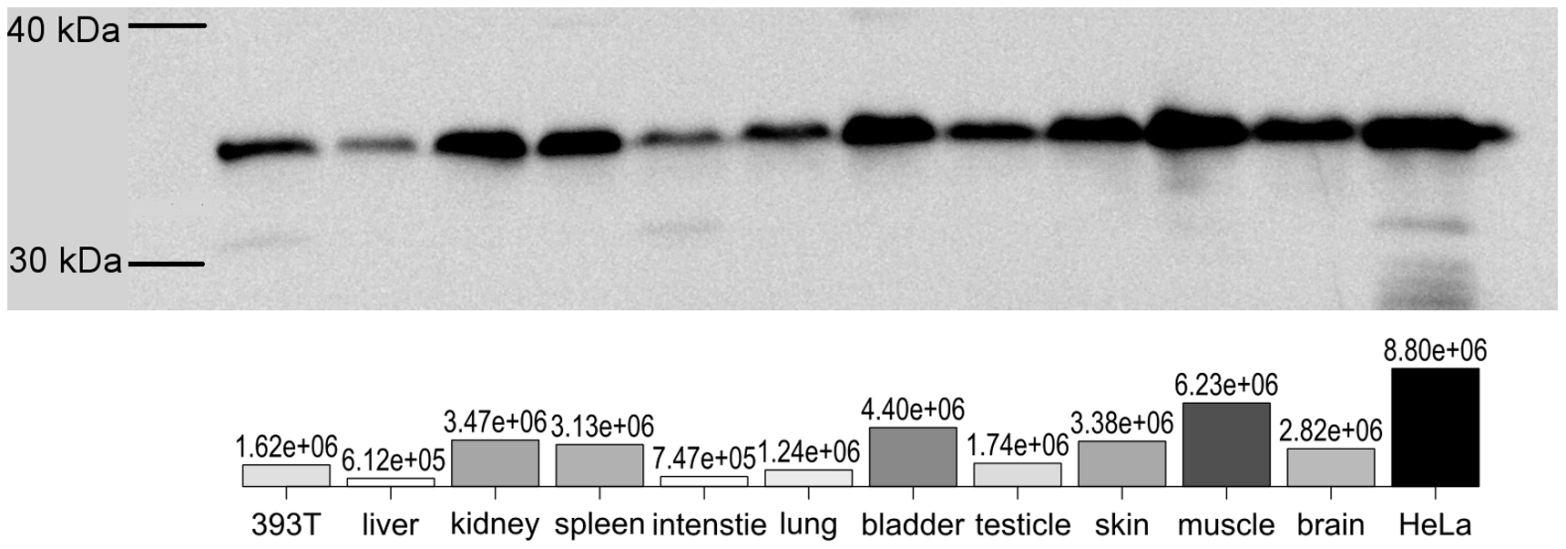
Examine tree shrew tissues with anti-human-GAPDH antibody. Upper: The blotting image, each lane load with 10 µg total protein. All bands in the same molecule weight level. Lower: each band's intensity value is shown below itself as bar and numeric.

The high structural homology (316 of 333 amino acids) lead to the possibility of identification of tree shrew's GAPDH molecule with anti-GAPDH antibody which reacted to human's. To assess the GAPDH protein expression, western blot analysis was performed to detect GAPDH using the anti-GAPDH antibody for human, that can recognize tree shrew GAPDH since our data showed that there was 95.2% homology of GAPDH between tree shrew and human (Table 1). All examined tissues were expressed GAPDH proteins (Fig.4). The high level expression tissues were kidney, spleen, bladder, skin, muscle and brain. And the lower expression tissues were liver and intestine.

## Discussion

In this study, we identified tree shrew's GAPDH full-length cDNA sequence, and performed evolution and sequence analysis base on GAPDH sequences. Our analyses are novel proofs to enhance the current classification of tree shrew: tree shrew is a small mammal which closer to human in phylogenetically relationship than other small experimental animals, such as mouse, rat, rabbit and guinea pig; and farther to human than chimpanzee, monkey, and pig. We also verified the cDNA sequence of tsGAPDH using qRT-PCR and verified the protein expression of tsGAPDH with western blot.

Tree shrew has small body size, relative low cost and relatively easy to use in the laboratories. Currently, tree shrew is classified as *Scandentia*, that is between *Primates* and *Insectivora*, though the very early studies suggest that the tree shrew has been grouped into the *Primates*. Our phylogenetic analysis supports the current classification for tree shrew. The phylogenetic relationship of tree shrew and human is closer than mouse, rat, guinea pig and rabbit. Thus, tree shrew can be used to closely mimic human disorders than use of mouse, rat, guinea pig or rabbit. The difference of GAPDH in amino acid's number between tree shrew and human is small. The number is only 17 of total 333 amino acids (Fig.2). We tried to analyze the cDNA sequence of tsGAPDH to find the tree shrew special sites but failed. Lack of tree shrew genome database and without well-understanding of the tree shrew genome, it was difficult to learn more genetic information based on only a cDNA sequence of housekeeping gene and a few genome information. Although it is enough to apply tsGAPDH as an internal reference in study with the tree shrew as experimental animal model.

GAPDH has been considered as a housekeeping gene in human, mouse, rat or other experimental animals. GAPDH is widely used as an internal reference in quantitative methods, including qRT-PCR, western blot, which are two popularly ones used to quantify RNA and protein content, respectively. In this study, tsGAPDH was verified that it widely and richly exists in each examined tissue of tree shrew by both qRT-PCR and western bolt. The tsGAPDH still can be measured even the sample was loaded in small quantity of 5 µg total protein by western blot; and if the quantity of total protein loading was more than 25 µg, it was difficult to analyze the difference between various tissues (unpublished data). But tsGAPDH was not expressed at an equal level in every tissue and it is not expressed in complete parallel level of RNA and protein. In our data (Fig.3 & Fig.4), tsGAPDH expressed in a significantly high level of RNA in skin among all measured tissues. Expression of tsGAPDH in muscle was lower than that in skin; and expression in kidney was lower than that in muscle. The RNA expressions in other tissues (liver, spleen, intestine, lung, bladder, testicle) were lower than that in kidney, and they were roughly equal between each other. The expression of tsGAPDH protein was different to RNA's. The tsGAPDH protein expressed in a high level in muscle, bladder, kidney, skin, spleen and brain. The liver and intestine had relatively lower content of tsGAPDH protein. In brief, tree shrew's muscle, kidney and skin had relatively high GAPDH expression level in both RNA and protein. The tissue preference of tsGAPDH maybe deserve more further works.

An ideal internal reference molecule should be stably expressed within all samples to be compared, regardless of tissue differences, experimental conditions, or treatments. Choosing an internal control gene to normalize gene expression data according to experiment's objective is one of the most crucial steps in the experimental design. Our data will be helpful references to choose internal reference molecule if target molecules expressed in different tree shrew's tissues/cells. It can be used to study both RNA and proteins. If treatments will be applied on samples, the reliability of GAPDH expression before and after treatment must be verified. According to our data, GAPDH is a suitable candidate just for comparing expression in different tissue those verified expressed in similar amount. These detecting primers for qRT-PCR and the verified anti-GAPDH antibody were good tools for tsGAPDH RNA and protein detection.

## Materials and Methods

### Ethics statement

All procedures related to animal subjects were reviewed and approved (permit numbers: GXUCM-20111203) by review committee of Guangxi University of Chinese Medicine, according with the Institutional Animal Care and Use regulations and rules.

### Tree shrew sampling

The tree shrews, belonging to subfamily of *Tupaia belangeri Chinensis*, were purchased from the Kunming Medical University. Three tree shrews (one male, two females) were used to collect blood and one male tree shrew sacrificed for tissue collection in this study. Animal care, blood collection, and tissues collection were conducted in Center of Experimental Animals, Guangxi University of Chinese Medicine.

### RACE and sequencing

Isolation of tsGAPDH cDNA sequence was carried out by 3′ and 5′ rapid amplification of cDNA ends (RACE) technology. In brief, total RNA was isolated from blood samples using the TRIzol Reagent (Life Technologies). The absorbance at 260 nm and 280 nm was measured using Take3 model in epoch plate reader (Biotek) to determine the concentration of RNA. First-strand cDNA was synthesized using the SMARTer RACE cDNA Amplification Kit (Clontech). Based on the published nucleotide sequences of the partial tree shrew GAPDH gene (Genbank AY699815.1), we designed 2 gene-specific primers (GSP) for 5′-RACE and 3′-RACE reaction, separately (Table 2). The 5′-RACE and 3′-RACE amplifications were preformed using *LA Taq* (TaKaRa). PCR products was electrophoresed on 1% agarose gel, and then purified using the Gel Extraction Kit (CWBio). Subsequently, RACE products were cloned into the pMD-18T Vector (TaKaRa) in accordance with established protocols, and then constructed plasmids were sequenced (Life Technologies).

### Sequence and phylogenetic Analysis

Alignment (Fig.2) and score (Table 1) was performed by clustalw2. The phylogenetic tree was compute and construct by the neighbor-joining method with 5000 bootstrap replicates [27]. Values greater than 50% were indicated. All GAPDH sequences used in this study are available in GenBank (Table S1).

### Organs and Tissues collection

Animal was anaesthetized with peritoneal injection of 10% Chloral Hydrate (0.2 ml/100 g). Warm physiological saline solution was injected through abdominal vein to expel those blood in organs. Organs of tree shrew were isolated after paled. And then organ was weighted, clipped to small tissues pieces, and separately sorted in RNAstore Reagent (CWBio) and Protease Inhibitor Cocktail Buffer (CWBio), which waiting to following total RNA and protein extraction process.

### Cells culture and collection

The 293T and HeLa cells were used as positive control according to the datasheet of anti-GAPDH antibody (GTX100118, GenTex). Cell lines cultured in RPMI 1640 Medium contain 10% FBS. Their collection was following the generally protocol. In brief, to detach cells form vessel with Trypsin-EDTA ((Life Technologies) and then wash with phosphate buffer solution. Collected cells proceed with protein extraction immediately.

### Construction of Standard Plasmid for Absolute qRT-PCR

Primers (Table 2) were designed and based on the sequence (GenBank KC215182) then synthesized (Life Technologies) in order to amplify the fragment to construct standard plasmid. Total RNA was isolated from blood samples as described before. The first-strand cDNA was synthesized using ProtoScript First Strand cDNA Synthesis Kit (New England Biolab Inc.). The fragment for standard plasmid was amplified with *LA Taq* (TaKaRa). PCR products was electrophoresed on 1% agarose gel, and purified using the Gel Extraction Kit (CWBio). Subsequently, purified PCR products were cloned into the pMD-18T Vector (TaKaRa) in accordance with established protocols. The constructed plasmid was proliferated in DH5α *E.coli* and then was extracted and purified with TIANprep Mini Plasmid Kit (TIANGEN). The absorbance at 260 nm and 280 nm was measured using Take3 model in Epoch Microplate Spectrophotometer (Biotek) to measure the concentration of the purified plasmid. Base on the concentration, purified plasmid was diluted to a serial of concentration gradient (10^11^, 10^10^, 10^9^, 10^8^, 10^7^ copies).

### qRT- PCR

Expression of tsGAPDH in various tissues of organs were measured with absolute qRT-PCR method following MIQE guidelines [16]. In brief, Total RNA was isolated with the TRIzol Reagent (Life Technologies) from isolated tissues those stored in RNAstore Reagent (CWBio). The absorbance at 260 nm and 280 nm was measured using Take3 model in Epoch Microplate Spectrophotometer (Biotek) to determine the concentration of the RNA. First-strand cDNA was synthesized using the PrimeScript II 1st Strand cDNA Synthesis Kit (TaKaRa). The qRT-PCR reactions were carried out using SYBR Premix Ex Taq II (TaKaRa) in Mastercycler ep realplex^4^ (Eppendorf). For each reaction, 2 µl first-strand cDNA and a final concentration of 50 nmol/L of each primer was used. The cycling profile consisted of an initial denaturation at 95°C for 5 min followed by 40 cycles of 95°C for 20 s, 55°C for 30 s, 72°C for 20 s, followed by melt curve analysis.

### Protein Extraction and Western Blotting

Protein extraction was processed after tissues collection and cell collection. The tissue protein was extracted with Tissue Protein Extraction Kit (CWBio) and cell protein was extracted with Mammalian Protein Extraction Kit (CWBio). Total protein was measured using Pierce BCA Protein Assay Kit (Thermo Scientific) on Epoch Microplate Spectrophotometer (Biotek). Sodium dodecyl sulfate polyacrylamide gel electrophoresis (SDS-PAGE) and western blotting was performed following the standard protocol. In brief, protein was loaded to SDS gel with amount of 10 µg/lane. After separated by electrophoresis, proteins were transferred from gel to nitrocellulose membrane under 120 V constant volt for 2 hours. Subsequently, membrane carried proteins was incubated in blocking buffer, washed, stained with anti-GAPDH antibody (GTX100118, GenTex), washed, stained with secondary HRP-conjugated IgG antibody (GTX213110-01, GenTex), successively. At last, the Pierce ECL Plus Western Blotting Substrate (Thermo Scientific) was used to expose blot in 4000 MM pro Image Station (Carestream). Experiments of western blot have been repeated more than five times and be executed by two independent operators, respectively.

### Data Analysis, Software and Statistics

Primers described in this article were designed with Primer3 online program [28], [29]. Clustalw2 online program [30] was used to align and score gene/protein sequences. BioEdit [31] was used to build alignment graph. MEGA [27] was used to compute and construct the phylogenetic tree. Nucleic acid's concentration was measured and calculated by Gen5 software (Biotek). Realplex software (Eppendorf) was used to analyze the qRT-PCR results. Western blotting graphs were captured and analyzed with Molecular Imaging Software (Carestream). Statistical comparison was carried out with One-Way ANOVA using agricolae package [32] in R [33]. Charts were built using ggplot2 [34], scales [35], and gridExtra [36] packages in R [33]. Charts and graphs were prepared with Inkscape [37] and GIMP [38].

## Supporting Information

Table S1 List of GAPDH sequence's Accession numbers in this study. These sequence were used to build phylogenetic tree or compare to tree shrew GAPDH in this study. All sequence is availed in GenBank database.
(DOC)

Result S1 **Statistical result.** Comparison of qRT-PCR in various tissues with one-way ANOVA followed by Duncan's Multiple Range Test.
(PDF)
