# Supplementary material for: Identification of Glyceraldehyde 3-Phosphate Dehydrogenase Sequence and Expression Profiles in Tree Shrew (Tupaia belangeri)
Source: PLoS One. 2014 Jun 2;9(6):e98552. doi: 10.1371/journal.pone.0098552 (PMC4041755; doi:10.1371/journal.pone.0098552)
Supplement: Table S1 — List of GAPDH sequence's Accession numbers in this study. These sequence were used to build phylogenetic tree or compare to tree shrew GAPDH in this study. All sequence is availed in GenBank database. (DOC) [file pone.0098552.s001.doc]

**Supplement 1. Accession numbers of GAPDH sequences** reported in this article

| Source | Accession number |
| --- | --- |
| Acartia pacifica | KC989857.1 |
| Acyrthosiphon pisum | AK343168.1 |
| Ailuropoda melanoleuca | FJ903446.1 |
| Anguilla japonica | AB075021.1 |
| Aplysia californica | KC417387.1 |
| Ascaris suum | AB058666.1 |
| Astatotilapia burtoni | AF123727.3 |
| Bactrocera dorsalis | GU269901.1 |
| Bemisia tabaci | KC161214.1 |
| Bombus hypocrita | JF751029.1 |
| Bombyx mori | DQ202316.2 |
| Bos grunniens | EU195062.1 |
| Bos taurus | BC102589.1 |
| Brachionus plicatilis | AB492236.1 |
| Brugia malayi | U18137.1 |
| Cancer borealis | HM157285.1 |
| Canis lupus familiaris | AB038240.1 |
| Cavia porcellus | EU862201.1 |
| Ceriodaphnia quadrangula | JX442203.1 |
| Cherax quadricarinatus | AY430092.1 |
| Clarias batrachus | KC414932.1 |
| Columba livia | AF036934.1 |
| Coptotermes formosanus | KC632253.1 |
| Cryptocercus punctulatus | JQ686947.1 |
| Danio rerio | BC083506.1 |
| Dermacentor variabilis | EU999993.1 |
| Dicentrarchus labrax | AY863148.1 |
| Dictyocaulus viviparus | EU152297.1 |
| Dirofilaria immitis | JQ780095.1 |
| Dugesia japonica | GU479914.1 |
| Echinococcus multilocularis | U19101.1 |
| Epinephelus coioides | GU982531.1 |
| Eptatretus stoutii | FJ462793.1 |
| Eriocheir sinensis | HM053701.1 |
| Esox lucius | BT079222.1 |
| Euscelidius variegatus | JX273239.1 |
| Felis catus | AB038241.1 |
| Gadus morhua | AY046595.1 |
| Gallus gallus | AF047874.1 |
| Glossina morsitans morsitans | DQ016434.1 |
| Haemonchus contortus | HM145749.1 |
| Haliotis discus discus | EF103374.1 |
| Helicoverpa armigera | JF417983.1 |
| Homalodisca vitripennis | AY588063.1 |
| Homo sapiens | AF261085.1 |
| Ictalurus furcatus | GU588044.1 |
| Ictalurus punctatus | GU588531.1 |
| Kryptolebias marmoratus | FJ438822.1 |
| Laodelphax striatella | HQ385974.1 |
| Lepeophtheirus salmonis | EF490852.1 |
| Liposcelis bostrychophila | FJ595241.1 |
| Locusta migratoria | JF915526.1 |
| Lysiphlebus testaceipes | DQ062222.1 |
| Macaca mulatta | NM_001195426.1 |
| Maconellicoccus hirsutus | EF070465.1 |
| Macrosteles quadripunctulatus | JX273237.1 |
| Meleagris gallopavo | GQ184819.1 |
| Meriones unguiculatus | AY066007.1 |
| Misgurnus anguillicaudatus | AB200264.1 |
| Mus musculus | GU214026.1 |
| Mustela putorius furo | EF392835.1 |
| Mythimna separata | HM055756.1 |
| Onchocerca volvulus | U96177.1 |
| Oncorhynchus mykiss | AB066373.1 |
| Oncorhynchus tshawytscha | AB177405.1 |
| Oreochromis niloticus | JN381952.1 |
| Oryctolagus cuniculus | L23961.1 |
| Osmerus mordax | BT074984.1 |
| Ostrea edulis | GQ150762.1 |
| Ovis aries | HM043737.1 |
| Pagrus major | AB069694.1 |
| Papilio polytes | AK402383.1 |
| Papilio xuthus | AK401207.1 |
| Paralichthys olivaceus | AB029337.1 |
| Pelteobagrus fulvidraco | JQ068865.1 |
| Periplaneta americana | JN411914.1 |
| Pongo abelii | NM_001132295.2 |
| Portunus trituberculatus | EU919707.1 |
| Procambarus clarkii | AB094145.1 |
| Rachycentron canadum | FJ825752.1 |
| Rana ridibunda | AY072703.1 |
| Rattus norvegicus | AF106860.2 |
| Reticulitermes flavipes | KC569743.1 |
| Schistocerca gregaria | HQ851387.1 |
| Schistosoma bovis | EU595761.1 |
| Schistosoma mansoni | XM_002576947.1 |
| Scylla paramamosain | JX268543.1 |
| Sparus aurata | DQ641630.1 |
| Spermophilus citellus | AY654895.1 |
| Spirometra erinaceieuropaei | AB031067.1 |
| Spodoptera frugiperda | KC262638.1 |
| Spodoptera litura | HQ012003.2 |
| Sus scrofa | AF017079.1 |
| Taenia solium | EF468494.1 |
| Takifugu rubripes | AB704200.1 |
| Trachemys scripta elegans | FJ514828.1 |
| Tupaia belangeri | KC215182 |
| Xenopus laevis | U41753.1 |
| Xenopus laevis Type B | AF549496.1 |
| Xenopus Silurana tropicalis | BC075438.1 |
