# Supplementary material for: Identification of Glyceraldehyde 3-Phosphate Dehydrogenase Sequence and Expression Profiles in Tree Shrew (Tupaia belangeri)
Source: PLoS One. 2014 Jun 2;9(6):e98552. doi: 10.1371/journal.pone.0098552 (PMC4041755; doi:10.1371/journal.pone.0098552)
Supplement: Result S1 — Statistical result. Comparison of qRT-PCR in various tissues with one-way ANOVA followed by Duncan's Multiple Range Test. (PDF) [file pone.0098552.s002.pdf]

Study: ANOVA Duncan test for GAPDH in tree shrew tissues

Duncan's new multiple range test
for copies

Mean Square Error: 6.12119e+15

Tissues, means

	copies	std.err	r	Min.	Max.
bladder	20187778	886957.6	9	1.77e+07	2.48e+07
intestine	12381111	644746.8	9	9.13e+06	1.51e+07
kidney	109171111	8334520.7	9	7.89e+07	1.49e+08
liver	13166667	434776.4	9	1.16e+07	1.56e+07
lung	34090000	2232486.2	9	2.22e+07	4.32e+07
muscle	198958444	10832455.1	9	1.49e+08	2.39e+08
skin	779920000	76953962.2	9	5.35e+08	1.32e+09
spleen	28465444	1724357.9	9	2.18e+07	3.92e+07
testicle	25878889	1767187.4	9	1.81e+07	3.44e+07

alpha: 0.05 ; Df Error: 72

Critical Range

2	3	4	5	6	7	8	9
73522453	77355740	79891503	81741679	83171782	84320165	85267657	86065389

Means with the same letter are not significantly different.

Groups, Treatments and means

a	skin	779900000
b	muscle	1.99e+08
c	kidney	109200000
d	lung	34090000
d	spleen	28470000
d	testicle	25880000
d	bladder	20190000
d	liver	13170000
d	intestine	12380000
